# Novel Therapeutics for Diabetic Retinopathy and Diabetic Macular Edema: A Pathophysiologic Perspective

**DOI:** 10.3389/fphys.2022.831616

**Published:** 2022-02-18

**Authors:** Katharine L. Bunch, Ammar A. Abdelrahman, Ruth B. Caldwell, R. William Caldwell

**Affiliations:** ^1^Department of Pharmacology and Toxicology, Medical College of Georgia, Augusta University, Augusta, GA, United States; ^2^James and Jean Culver Vision Discovery Institute, Medical College of Georgia, Augusta University, Augusta, GA, United States; ^3^Department of Cellular Biology and Anatomy, Medical College of Georgia, Augusta University, Augusta, GA, United States; ^4^Vascular Biology Center, Medical College of Georgia, Augusta University, Augusta, GA, United States

**Keywords:** diabetic retinopathy, diabetic macular edema, arginase, erythropoietin, nitric oxide, antioxidant, inducible nitric oxide synthase

## Abstract

Diabetic retinopathy (DR) and diabetic macular edema (DME) are retinal complications of diabetes that can lead to loss of vision and impaired quality of life. The current gold standard therapies for treatment of DR and DME focus on advanced disease, are invasive, expensive, and can trigger adverse side-effects, necessitating the development of more effective, affordable, and accessible therapies that can target early stage disease. The pathogenesis and pathophysiology of DR is complex and multifactorial, involving the interplay between the effects of hyperglycemia, hyperlipidemia, hypoxia, and production of reactive oxygen species (ROS) in the promotion of neurovascular dysfunction and immune cell polarization to a proinflammatory state. The pathophysiology of DR provides several therapeutic targets that have the potential to attenuate disease progression. Current novel DR and DME therapies under investigation include erythropoietin-derived peptides, inducers of antioxidant gene expression, activators of nitric oxide/cyclic GMP signaling pathways, and manipulation of arginase activity. This review aims to aid understanding of DR and DME pathophysiology and explore novel therapies that capitalize on our knowledge of these diabetic retinal complications.

## Introduction

Diabetic retinopathy (DR) is a feared complication of diabetes that dramatically affects quality of life through vision deterioration and loss (Hartnett et al., 2017). DR develops with prolonged hyperglycemia, which can occur with both type 1 diabetes mellitus (T1DM) and type 2 diabetes mellitus (T2DM) (Fong et al., 2004). According to the World Health Organization, the incidence of diabetes increased by approximately 290% between 1980 and 2014, and the frequency of diabetes-related premature mortality is climbing (WHO, 2021). The increased global prevalence of diabetes has resulted in rampant DR, the leading cause of blindness in working-age adults (WHO, 2021). Not only does DR affect patients individually, but it also represents a significant healthcare and economic burden (WHO, 2021).

Hyperglycemia, hyperlipidemia, and formation of reactive oxygen species (ROS) initiate and perpetuate many processes involved in the pathogenesis of DR. Clinically, DR is divided into two primary stages: non-proliferative and proliferative DR (Fong et al., 2004; Hartnett et al., 2017; Kern et al., 2019). Non-proliferative DR can be further classified into mild, moderate, severe, and very severe (Duh et al., 2017). Proliferative DR (PDR) is differentiated from non-proliferative DR by the presence of neovascularization, and can be stratified into early, high-risk, or severe, based on location and quantity of neovascularization, presence of preretinal or intravitreal hemorrhage, and macular or retinal detachment (Duh et al., 2017). Diabetic macular edema (DME), a vision-threatening complication of both non-proliferative and PDR, results from fluid extravasation through damaged and pathologic microvasculature (Gupta et al., 2013; Duh et al., 2017). Though the etiologies of T1DM and T2DM differ, the overlapping characteristics of hyperglycemia, hyperlipidemia, hypoxia, and formation of ROS, provide the milieu necessary for development and progression of DR and DME (O’Brien et al., 1998; Gylling et al., 2004; Kahn et al., 2006).

## Pathophysiology of Diabetic Retinopathy and Diabetic Macular Edema

### Hyperglycemia

Type 1 diabetes mellitus and T2DM represent a spectrum of diseases whose presentation and progression can vary considerably, yet they are unified by the predominant feature, hyperglycemia (American Diabetes Association, 2021). While T1DM is characterized by hyperglycemia secondary to lack of endogenous insulin production, T2DM is associated with systemic insulin resistance and is associated with metabolic syndrome, a cluster of metabolic derangements including hypertension, increased central adiposity, and hypertriglyceridemia (Samson and Garber, 2014; Katsarou et al., 2017). Sustained hyperglycemia results in several biochemical, metabolic, and vascular abnormalities that are responsible for disease progression, such as production of advanced glycosylation end-products (AGEs) and activation of the protein kinase C (PKC), polyol, and hexosamine pathways, all of which can promote increased production of cytokines and growth factors (Urias et al., 2017; Ighodaro, 2018).

AGEs are formed *via* a three-step process initiated by high blood glucose (Ott et al., 2014; Singh et al., 2014). When formed, AGEs create cross-linked, non-degradable aggregates of proteins, lipids, and nucleic acids (Ott et al., 2014; Singh et al., 2014). Cross-linking of the extracellular matrix causes stiffening of the vasculature and promotes organ dysfunction (Ott et al., 2014; Singh et al., 2014). Thus, AGE aggregation can compromise protein, lipid bilayer, and collagen function (Ott et al., 2014; Singh et al., 2014). Additionally, these aggregates initiate inflammatory, angiogenic, vascular remodeling, and apoptosis signal cascades *via* several receptors (Ott et al., 2014). These signal cascades are integral in the pathogenesis of diabetes, DR, and DME, inducing vascular dysfunction and remodeling, angiogenesis, thrombogenesis, atherosclerosis, and hypoxia (Li et al., 2004; Chang et al., 2008; Yamagishi, 2011; Hegab et al., 2012; Roy, 2013). When AGEs bind to the receptor for AGEs (RAGE), a signal cascade promotes angiogenesis through upregulation of vascular endothelial growth factor (VEGF), a key mediator of neovascularization in PDR (Li et al., 2004; Chang et al., 2008; Yamagishi, 2011; Roy, 2013; Ott et al., 2014; Singh et al., 2014). New vessel formation in the hypoxic and inflammatory milieu of DR is aberrant and promotes DME development, as these fragile retinal vessels have poor integrity, leading to widespread fluid extravasation (Gupta et al., 2013; Urias et al., 2017).

In addition to promoting AGE formation, hyperglycemia activates the polyol pathway, which metabolizes glucose under conditions of glucose excess and produces sorbitol *via* aldose reductase with oxidation of NADPH to NADP^+^ (Ighodaro, 2018; Kang and Yang, 2020). Most tissues in the body have sorbitol dehydrogenase which converts sorbitol to fructose *via* reduction of NAD^+^ to NADH, allowing for progression of this metabolic pathway. However, sorbitol dehydrogenase is not readily expressed in the retina, resulting in a build-up of osmotically active sorbitol, leading to retinal dysfunction (Thul et al., 2017; Kang and Yang, 2020). Consumption of NADPH in the polyol pathway impairs antioxidant mechanisms and increases vulnerability of retinal tissues to oxidative stress.

The hexosamine pathway metabolizes glucose under conditions of hyperglycemia. The end-product of this pathway, uridine diphosphate N-acetylglucosamine (UDP-GlcNAc), catalyzes the addition of O-GlcNAc moieties to serine and threonine residues of proteins, altering their function and resulting in retinal damage (Semba et al., 2014). Additionally, activation of the hexosamine pathway results in upregulation of cholesterol synthesis through O-GlcNAcylation and activation of cholesterol transcription factor, specificity protein 1 (Sp1), leading to hypercholesterolemia, which independently contributes to disease progression (Semba et al., 2014). Although activation of the AGE-RAGE, polyol, and hexosamine pathways initiate a series of processes directly involved in the pathogenesis of DR, hyperlipidemia and formation of reactive oxygen species (ROS) also strongly contribute to disease progression.

### Hyperlipidemia

Both T1DM and T2DM are associated with a hyperlipidemic state (Shah et al., 2017). Due to the development of insulin resistance in T2DM and the lack of exogenous insulin production in T1DM, the cellular uptake of glucose is blunted despite high blood glucose levels. This state of relative starvation promotes lipolysis and cholesterol accumulation, resulting in elevated blood lipids (Lopaschuk, 2016). Hyperlipidemia not only contributes to systemic disease progression, but also to progression of DR and DME. Accumulation of lipid particles on endothelial cells (EC) induces vascular damage and impairs regional blood flow, promoting a state of retinal hypoxia (Tetè et al., 2012). The vascular damage from lipid accumulation contributes to breakdown of the blood-retinal-barrier (BRB). This increased vascular permeability predisposes to DME development and damage to the retinal neurovascular unit (Urias et al., 2017). Additionally, vascular lipid accumulation activates leukocytes, which secrete chemokines to promote inflammatory macrophage infiltration into the retina and to induce polarization of retinal microglia to a pro-inflammatory phenotype (Tetè et al., 2012; Kinuthia et al., 2020). Activated macrophages and microglia sustain the inflammatory state through secretion of cytokines that compromise the BRB and contribute to VEGF production, which exacerbates the BRB breakdown and promotes angiogenesis and neovascularization (Tetè et al., 2012).

Additionally, hyperlipidemia accelerates hyperglycemia-induced mitochondrial damage, further promoting an inflammatory state (Rao et al., 2021). Hyperlipidemia has also been shown to result in increased intracellular calcium and pro-inflammatory cytokine production, which increases the expression of inducible nitric oxide synthase (iNOS), the NOS isoform that produces cytotoxic quantities of nitric oxide (NO) as an immune defense mechanism (Bogdan, 2015; García-Ortiz and Serrador, 2018; Rao et al., 2021).

### Reactive Oxygen and Nitrogen Species

The formation of ROS and reactive nitrogen species (RNS) and the subsequent damage caused by these volatile molecules is intimately linked to the pathogenesis of DR, playing an essential role in disease progression by amplifying the ischemic and inflammatory milieu. ROS and RNS, such as superoxide, hydrogen peroxide, hydroxyl radicals, and peroxynitrite, are formed *via* several mechanisms. One mechanism is *via* a hyperglycemia-induced increase in activity of NADPH oxidases, which results in superoxide or hydrogen peroxide formation (Kowluru, 2021). Another mechanism is through uncoupling of NOS, *via* reduced L-arginine availability, which forms superoxide instead of producing NO (Roe and Ren, 2012). The consequences of NOS uncoupling are twofold, with impaired vasodilation resulting in hypoxia and superoxide production leading to inflammation and tissue dysfunction (Guzik et al., 2003). The hypoxic state further accentuates formation of ROS, vascular dysfunction, VEGF production, and inflammation *via* downstream effectors of hypoxia-inducible factor isoforms (Gunton, 2020). Hyperglycemia increases PKC activation, which plays a critical role in the formation and amplification of ROS. It has also been demonstrated that PKC augments NADPH oxidase activity, further contributing to oxidative stress (Kang and Yang, 2020). Accumulation of ROS not only contributes to the vascular and metabolic dysfunction in DR, but also promotes formation of AGEs and increases polyol pathway flux, exacerbating the damage.

## The Need

With the epidemic-level increase in cases of diabetes, more individuals are at risk of DR-induced vision loss, necessitating the development of more effective, affordable, and accessible treatment modalities (Urias et al., 2017; Schmidt, 2018). The current gold-standard therapies for DR and DME are pan-retinal photocoagulation (PRP) and intravitreal injections of anti-VEGF or steroid drug formulations (Funatsu et al., 1996; Gupta et al., 2013). PRP involves laser ablation of the outer retina, which is thought to slow progression of DR by destroying peripheral tissue with high oxygen demand, thereby diverting oxygen to the ischemic central retina and decreasing hypoxia-induced release of inflammatory cytokines and VEGF (Funatsu et al., 1996). Though this method is efficacious in blunting progression of DR, it is associated with notable complications including visual field impairment, night vision deficits, and suprachoroidal and macular effusions that promote exudative retinal detachment (Reddy and Husain, 2018). Anti-VEGF intravitreal therapies target the hypoxia-induced expression of VEGF, attenuating neovascularization in PDR and breakdown of the BRB in DME (Gupta et al., 2013). Though this treatment can be effective in many patients, some DME patients are treatment-resistant or exhibit only transient improvement (Urias et al., 2017). Intravitreal corticosteroid injections target retinal inflammation but can promote VEGF expression (Schwartz et al., 2016). Intravitreal injections require frequent office visits and are associated with poor patient compliance due to fear of intravitreal administration, financial limitations, and perceived lack of efficacy (Polat et al., 2017). Moreover, intravitreal injections are associated with significant risks (Sachdeva et al., 2016; Tan et al., 2021). With each injection, patients are at risk of developing acute exogenous endophthalmitis, a serious medical emergency characterized by bacterial or fungal infection of the vitreous and/or aqueous humor (Day et al., 2011; Sachdeva et al., 2016; Durand, 2020). Anti-VEGF injections also increase the risk of tractional retinal detachment, elevations in intraocular pressure, and uveitis (Heier et al., 2012).

The developing understanding of the neurovascular interactions in health and disease is guiding efforts to find novel therapeutic targets capable of arresting DR at early stages (Simo et al., 2021). Strategies for promoting tissue repair and preventing neuroinflammation have shown promising results in the treatment of neurovascular diabetic complications. The discovery of erythropoietin (EPO) production within retinal tissue has prompted investigations into its physiologic roles and therapeutic potential (Brines et al., 2008). Distinct from its role in hematopoiesis, EPO has been found to mediate neuroprotective effects in the retina, resulting in development of EPO peptide derivatives (Brines et al., 2008). ARA290 is a peptide that binds to the EPO innate repair receptor but lacks the erythrogenic activity of endogenous EPO (Brines et al., 2015). ARA290 has shown potential therapeutic benefit in minimizing glial activation, neuronal loss, and promoting tissue repair in preclinical studies of DR (McVicar et al., 2011). In clinical trials, ARA290 administration in diabetic patients blunted metabolic dysfunction and symptoms of diabetic neuropathy (Brines et al., 2015). Another novel therapeutic approach targeting early stages of DR and DME is NO/cyclic GMP (cGMP) modulator drug therapy, which capitalizes on the various roles of NO signaling in DR pathogenesis. Runcaciguat, a novel cGMP activator, is undergoing clinical trials to assess its efficacy in both diabetic nephropathy and DR (Hahn et al., 2021). Although clinical trials with antioxidant and radical scavenging molecules have failed to show sufficient protection from DR progression, strategies for activating tissue antioxidant mechanisms have shown great therapeutic promise in retinopathies involving oxidative stress (Nakagami, 2016; Evans and Lawrenson, 2017). Nuclear factor erythroid 2-related factor 2 (Nrf2) is a novel potential target that upregulates transcription of antioxidant proteins and has shown great promise in the treatment of retinal diseases (Nakagami, 2016). Finally, our group has demonstrated the complex roles of the arginase isoforms in the development and progression of DR, illustrating the potential therapeutic benefit of targeting these enzymes in diseases that impact the neurovasculature (Patel et al., 2013; Caldwell et al., 2015; Shosha et al., 2016, 2021; Atawia et al., 2020).

## Arginase and Diabetic Retinopathy

### Arginase as a Therapeutic Target

Diabetes increases the expression of arginase isoforms, which have a crucial role in the development of diabetes-induced complications (Patel et al., 2013). Arginase, the key enzyme in the hepatic urea cycle, reciprocally regulates NO production by competing with NOS for their shared substrate, L-arginine (Elms et al., 2013; Caldwell et al., 2015; Wang et al., 2015). This ureohydrolase has two isoforms, arginase 1 (Arg1), which is cytoplasmic and highly expressed by hepatocytes, and arginase 2 (Arg2) which is primarily localized to the mitochondria. Both isoforms are found in a variety of cells, including retinal, endothelial, neuronal, and immune cells. Additionally, both can be upregulated under conditions of hyperglycemia, inflammation, and increased ROS (Romero et al., 2008; Chandra et al., 2012; Pandey et al., 2014; Caldwell et al., 2018). Arg1 and Arg2 are expressed in various layers and cell types of the retina (Patel et al., 2013). Arg1 immunoreactivity is prominent in neuronal cells within the ganglion cell layer, inner nuclear layer, and in Müller glial cells. Pronounced Arg2 immunoreactivity is evident in cells of the inner nuclear and nerve fiber layers as well as in horizontal cells (Narayanan et al., 2013; Patel et al., 2013). However, given the mitochondrial localization of Arg2, it is likely that a basal level of Arg2 is present in all cell types.

Increased arginase expression leads to elevated levels of L-ornithine, a downstream product of arginase activity. The metabolism of L-ornithine by ornithine decarboxylase (ODC) and ornithine aminotransferase results in the production of polyamines (putrescine, and downstream products spermidine and spermine), and proline and glutamate, respectively (Caldwell et al., 2018). Production of proline is necessary for collagen formation, which contributes to wound healing and fibrosis (Wu and Morris, 1998; Caldwell et al., 2018). Polyamines are important in cell proliferation, ion channel function, and neuroprotection (Narayanan et al., 2013; Caldwell et al., 2015). Activation of the ODC pathway has been shown to limit inflammation, reduce iNOS activation, and promote a reparative M2-like macrophage phenotype by increasing the production of putrescine in a model of bacterial infection (Hardbower et al., 2017). However, the reverse activity of this pathway (spermine to spermidine to putrescine), generates hydrogen peroxide and toxic acrolein, which can exacerbate the pathology (Narayanan et al., 2014, 2019; Alfarhan et al., 2020).

### Arginase 1 and Vascular Tone

The competition between arginase and NOS for L-arginine is of particular importance in vascular tone regulation given the crucial role of NO signaling in vasodilation, leukocyte adhesion, and platelet aggregation (Caldwell et al., 2018). In fact, elevated Arg1 is involved in endothelial dysfunction in a wide range of vascular beds including pulmonary, coronary, aortic, and mesenteric vasculature (Beleznai et al., 2011; Lucas et al., 2013; Toque et al., 2013; Kövamees et al., 2016; Caldwell et al., 2018). In retinal blood vessels, increased Arg1 immunoreactivity was detected as early as 8 weeks in diabetic mice (Elms et al., 2013). Furthermore, partial deletion of Arg1 or treatment with the arginase inhibitor, 2(S)-amino-6-boronohexanoic acid (ABH), resulted in improved EC-mediated vasodilation in the retinal vasculature of diabetic murine models (Elms et al., 2013). Together, these results suggest that Arg1 plays a role in diabetes-induced endothelial dysfunction in retinal vasculature.

### Arginase 1 in Retinal Ischemia

In DR, damage to the retinal microvasculature leads to impaired tissue perfusion and subsequent hypoxia, which is further accentuated by increased leukocyte adhesion (Cai and Boulton, 2002; van der Wijk et al., 2017). Despite the prominent role of Arg1 in diabetes-induced endothelial dysfunction, a neuroprotective role of Arg1 has been surprisingly revealed in murine models of retinal ischemia/reperfusion injury (I/R) (Malek et al., 2018). In this model, mice with either partial global deletion of Arg1 (Arg1^±^) or myeloid-specific Arg1 deletion demonstrated significantly worse neuronal and microvascular degeneration compared to their littermate controls. However, this effect was not seen in EC-specific Arg1 knockout mice (Fouda et al., 2018). It is postulated that the neuroprotective effects of Arg1 are due to the reduced availability of L-arginine for iNOS, which functions to amplify the pro-inflammatory state through increased macrophage polarization to the M1-like, pro-inflammatory phenotype (Lee et al., 2017). Elevated Arg1 reduces L-arginine levels, the substrate necessary for iNOS to maintain high NO production and inflammation. In fact, control mice subjected to I/R exhibited fewer macrophages in the M1-like, pro-inflammatory state (Fouda et al., 2018). In acute hypoxic injury, such as I/R, these anti-inflammatory, neuroprotective effects of Arg1 seem to outweigh the detrimental effects of Arg1 on the vascular endothelium (Fouda et al., 2018, 2020). Intravitreal and intraperitoneal treatment with pegylated Arg1 (PEG-Arg1), (Arg1 covalently linked to polyethylene glycol with a prolonged half-life), in murine models of I/R exhibited significant protection against retinal neurodegeneration (Fouda et al., 2018, 2022). Macrophages pretreated with PEG-Arg1 *in vitro* also exhibited reduced cytokine production and iNOS expression after exposure to bacterial lipopolysaccharide (LPS). Additionally, PEG-Arg1 treatment of these macrophages post-LPS exposure reversed the LPS-induced mitochondrial dysfunction (Fouda et al., 2018). The neuroprotective effects of PEG-Arg1 are likely secondary to blockade of iNOS-induced oxidative stress, blunting perpetuation of inflammation and oxidation by retinal macrophages and microglia.

### Arginase 1 and Immune Modulation

The role of Arg1 in the polarization of macrophages toward a reparative, M2-like phenotype and its effects on other immune cell function has been extensively studied, however, the mechanisms are not entirely understood (Kieler et al., 2021). As briefly discussed, L-arginine depletion by Arg1 has been proposed as a mechanism that mediates decreased production of cytotoxic NO levels and blunts iNOS perpetuation of inflammation and oxidative stress (Lee et al., 2003). Additionally, L-arginine depletion results in T-lymphocyte dysfunction through inhibition of T-cell receptor CD3ζ chain expression (Rodriguez et al., 2003). Another proposed mechanism for Arg1 modulation of immune cell responses is through the Arg1-induced production of polyamines *via* the ODC pathway, which has been shown to promote the reparative M2-like phenotype in macrophages and microglia (Van den Bossche et al., 2012; Latour et al., 2020). Independent of Arg1 expression, production of polyamines was necessary for M2-like polarization in cultured macrophages stimulated with IL-4 (Van den Bossche et al., 2012). Additionally, depletion of polyamines in cultured macrophages resulted in the amplification of LPS-induced inflammatory gene expression (Van den Bossche et al., 2012). Another study found that elevated levels of putrescine attenuated macrophage polarization to the pro-inflammatory M1-like phenotype and proposed that this effect was *via* histone modifications (Hardbower et al., 2017). Though these are some possible explanations, more studies are necessary to unravel the seemingly multifaceted effects of Arg1 on immune cell modulation.

### Arginase 2 and Diabetic Retinopathy

In contrast to Arg1, global or EC-specific deletion of Arg2 in mice results in reduced oxidative and nitrative stress, decreased neurovascular damage, and absence of glial activation in the retina after I/R injury (Shosha et al., 2016, 2021). In both the I/R *in vivo* model and in EC exposed to oxygen and glucose deprivation/reperfusion (OGD/R), Arg2 mRNA and protein expression were markedly increased (Shosha et al., 2016, 2021). Furthermore, overexpression of Arg2 in EC subjected to OGD/R resulted in increased mitochondrial dysfunction and fragmentation, and amplified cell stress and apoptosis (Shosha et al., 2021). Interestingly, pan-arginase inhibition in EC with ABH resulted in inhibition of OGD/R-induced cell stress but did not promote cell survival. This lack of survival with pan-arginase inhibition is postulated to be due to the loss of Arg1-mediated protective effects. Mice with a global Arg2 deletion challenged by a high-fat, high-sucrose diet were protected against retinal oxidative stress, inflammasome activation, and pro-inflammatory activation of retinal glial cells, indicating that Arg2 plays a role in the early pathogenesis of DR (Atawia et al., 2020). The apparent dichotomy of these arginase isoforms under various pathologic conditions warrants the development of therapies targeted to either specific inhibition of Arg2 or selective delivery of Arg1 to macrophages.

## Conclusion

With the prevalence of diabetes on the rise worldwide, more people are at an increased risk of developing vision-compromising DR and DME, necessitating the development of more efficacious and patient-friendly treatment modalities. DM is a systemic, multifactorial disease that involves a multitude of interconnected processes. Studies examining the nuances of the pathogenesis and pathophysiology of DR and DME have elucidated many promising novel therapeutic targets. EPO signaling in tissue repair, cGMP activation, and activation of antioxidant gene transcription are promising therapeutic targets that can modify disease progression and can be utilized at earlier disease stages (Brines et al., 2008, 2015; Nakagami, 2016; Evans and Lawrenson, 2017; Hahn et al., 2021). Capitalizing on Arg1 and Arg2 activity in the disease pathogenesis of DM also shows therapeutic potential. PEG-Arg1 has demonstrated a neuroprotective effect in ischemic retinopathy that appears to outweigh the detrimental effect on the vasculature by attenuating production of inflammatory cytokines through suppression of iNOS activity (Caldwell et al., 2018; Fouda et al., 2018, 2022). Deletion of Arg2 has exhibited protective effects through the attenuation of inflammasome activation and polarization of macrophages and retinal microglia (Narayanan et al., 2014; Shosha et al., 2016; Atawia et al., 2019, 2020). There is hope that these novel therapies will prove to be effective in attenuating DR and DME progression, thereby decreasing the number of individuals at risk of diabetes-induced vision loss.

## Author Contributions

KLB and RWC conferred on the theme and general content. KLB along with AAA drafted the sections of the manuscript. RBC and RWC provided guidance and editorial feedback. All authors contributed to the article and approved the submitted version.

## Conflict of Interest

The authors declare that the research was conducted in the absence of any commercial or financial relationships that could be construed as a potential conflict of interest.

## Publisher’s Note

All claims expressed in this article are solely those of the authors and do not necessarily represent those of their affiliated organizations, or those of the publisher, the editors and the reviewers. Any product that may be evaluated in this article, or claim that may be made by its manufacturer, is not guaranteed or endorsed by the publisher.
